# Influence of Column Axial Load and Heat Affected Zone on the Strength of Aluminium Column Web in Tension

**DOI:** 10.3390/ma7053557

**Published:** 2014-05-06

**Authors:** Gianfranco De Matteis, Gianluca Sarracco, Giuseppe Brando, Federico M. Mazzolani

**Affiliations:** 1 Department of Engineering and Geology, University “G. d’Annunzio” of Chieti-Pescara, Pescara 65127, Italy; E-Mails: g.sarracco@unich.it (G.S.); gbrando@unich.it (G.B.); 2 Department of Structure for Engineering and Architecture, University “Federico II” of Naples, Naples 80125, Italy; E-Mail: fmm@unina.it

**Keywords:** connections, joints, aluminium, column web in tension, axial load, heat affected zone

## Abstract

The component method for aluminium joints has been recently introduced in some codes and guidelines. Nevertheless, it is still in need of some development and improvement, as in some cases it was obtained by adapting the existing formulations that are valid for steel. The current paper presents the main outcomes of a parametric analysis carried out by means of finite element (FE) numerical models for determining the influence of both column axial load and heat affected zone—in the case of welded details—on the structural response of the column web in a tension component. The proposed study integrates previous research carried out by the authors, where the influence of the assumed alloy was investigated and interpreted by corrective parameters expressed as a function of both the material strain hardening and ductility.

## 1. Introduction

The prediction of the behaviour of aluminium connections is a worthwhile topic, under development since many decades. With particular regard to beam-to-column joints, for analysis purposes, it would be important to have the possibility to use a moment-rotation (M-Φ) relationship to describe both the linear and non-linear response of the connection. This could be determined by experimental tests and, alternatively, by validated analytical methods, such as the component method, which is currently used for steel.

At this stage, codes and guidelines dealing with aluminium structures, such as EC9 [1] and the Italian guidelines CNR-DT 208 [2], propose the application of the above method, which is provided in a simplified way, by applying some correction coefficients to the formulations given by EC3 [3] for steel joints. In fact, the correctness of this assumption was proven by detailed studies but dealing with only some specific components, namely the T-stub [4–6]. Additional researches are therefore necessary for covering the other typical joint components, taking into account that aluminium alloys, contrarily to steel, already exhibit σ-ε non-linear behaviour for low deformation levels. In addition, the post-elastic behaviour is normally characterized by both a significant strain hardening and a limited ductility, having an influence on the ultimate strength as well as on the development of the resisting mechanism in plastic range [7].

The current paper, which is framed as a research activity aimed at providing more reliable formulations for the interpretation of the behaviour of aluminium connections, investigates a typical component in a beam-to-column joint, namely the column web subjected to tension forces. A parametric analysis is carried out, by means of a finite element (FE) numerical model, with the aim of investigating the interaction between the component strength and the column axial force. In addition, the reduction of strength due to the presence of the heat affected zone is studied.

On the basis of the obtained results, new formulations are provided for possible implementation in codes and recommendations.

## 2. Literature Overview

Apart from possible buckling phenomena, the components corresponding to column web in tension and compression are characterized by the same behaviour. This aspect allows retrieving a large number of studies in literature, which are the basis of current formulations adopted in the EC3 for steel joints.

For welded steel beam-to-column joints, a significant contribution was provided in [7–9]. In particular, in the latter the following formulation for the strength of a steel column web in compression (*F*_cwc,Rd_) was established:

$$F_{\text{cwc,Rd}}=f_{y}t_{\text{wc}}b_{\text{eff}}=f_{y}t_{\text{wc}}\left[t_{\text{fb}}+2\sqrt{2}\;a+5(t_{\text{fc}}+r_{c})\right]$$ (1)

In the above equation, *t*_wc_ and *t*_fc_ are the column web and flange thicknesses, respectively, *t*_fb_ is the beam flange thickness, *r*_c_ the column fillet root radius, *a* the throat thickness of the fillet weld connecting the beam flange to the column one and *f*_y_ is the specific yielding strength.

The expression in the square bracket represents the effective width *b*_eff_, which is conventionally the extension, along the column longitudinal direction, of the part of column web involved in the failure mechanism.

**Figure 1.**
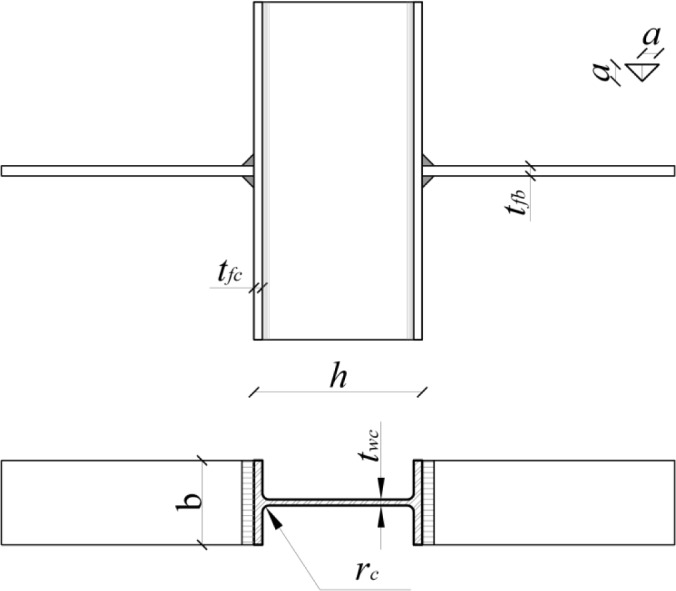
Representation of the geometrical configuration of the specimens tested by Matusiak et al. [10].

With regard to aluminium beam-to-column joints, experimental and numerical studies, aimed at predicting the yielding strength of column web in tension, have been carried out by Matusiak [10] at the Norwegian University of Science and Technology of Trondheim (NTNU), where specimens made of the EN AW 6082-T6 alloy and characterized by the geometry depicted in Figure 1, a I-100 section profile, were investigated.

The failure mode observed experimentally consisted in a column web fracture similar to that expected for steel. For this reason, the following elastic strength was proposed (Equation (2)):

$$F_{\text{cwt,el}}=t_{\text{wc}}b_{\text{ eff,al}}f_{0.2}$$ (2)

As a consequence, the same base formulation fixed for steel in Equation (1) was assumed, but the yielding strength of steel was replaced with the conventional elastic strength of aluminium (*f*_0.2_). On the other hand, the effective width *b*_eff,al_ was assumed according to Equation (3), which is different by the one proposed for steel, due to a different development of the plastic mechanism, because of the aluminium strain hardening and limited ductility.

$$b_{\text{eff},\text{el}}=t_{\text{fb}}+2\sqrt{2}a+3.4(t_{\text{fc}}+r_{\text{c}})$$ (3)

On the basis of the above experimental tests, a numerical FE model has been set up and presented by the authors in [11]. It has been employed in order to carry out parametric analysis for investigating the influence of the used alloy mechanical features on the strength *F*_Rd,0_ of the column web in tension component. It has been found that this can be interpreted conservatively by the following expression (Equation (4)).

$$F_{\text{Rd},0}=f_{u}\cdot t_{\text{wc}}\cdot b_{\text{eff,alu}}=f_{u}\cdot t_{w}\cdot \left[t_{\text{pl}}+2\sqrt{2}a_{b}+2\beta (t_{\text{fc}}+r_{c})\right]$$ (4)

The above formulation takes into account that the resisting mechanism changes, at the variation of the base material, according to a β factor that depends on the ultimate strain ε_u_ and the strain hardening parameter *n*_p_ of the associated Ramberg-Osgood relationship [12], as provided by Equations (5)–(7).

$$\beta \left(\varepsilon_{u},n_{p}\right)=\xi \left(\varepsilon_{u}\right)\cdot \eta \left(n_{P}\right)$$ (5)

where

$$\xi \left(\varepsilon_{u}\right)=0.21\cdot \ln\left(\varepsilon_{u}-1.6\right)+2.2$$ (6)

and

η(np)=min[1.00;0.31⋅ln(np23−2)+2.232.86] (7)

**Figure 2.**
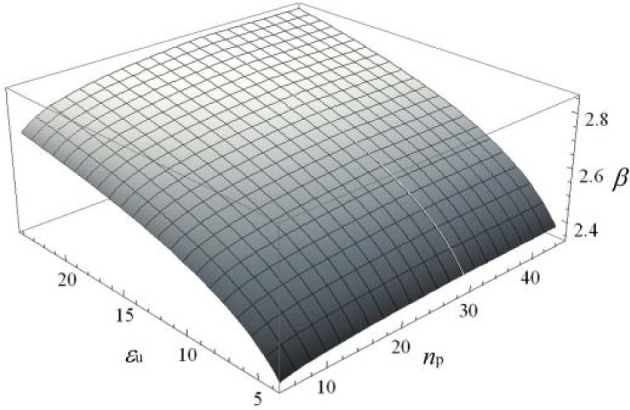
The β correction factor of the effective width as a function of ε_u_ and *n*_p_.

The function given in Equation (5) is also plotted in Figure 2.

## 3. The Proposed FE Model

**Figure 3.**
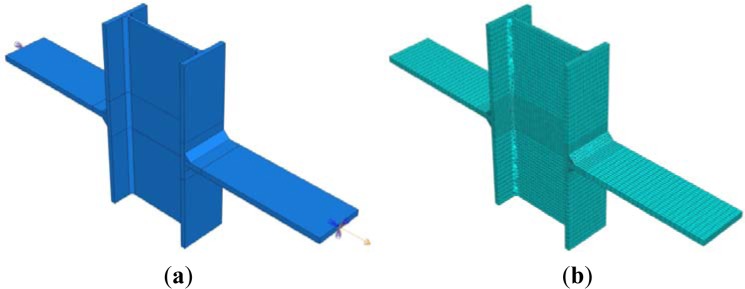
The proposed FE numerical model: (a) geometry and (**b**) assigned mesh.

The geometry of the plate-to-column specimens tested by Matusiak [11] has been modelled (see Figure 3a) by using the ABAQUS CAE non-linear software [13]. The three-dimensional eight-node hexahedral finite C3D8R elements, with reduced integration, have been adopted for meshing all the parts, except the ones representing the weld seams. For the latter, the four-node linear tetrahedral elements (C3D4) have been used in order to prevent hourglass control problems (see Figure 3b). Particular care has been spent for the meshing algorithm: three elements have been set along the column flanges thickness, while two elements have been imposed transversally to the column web. A more refined mesh has been imposed in the proximity of the plate-to-column flange connection, in order to account for the higher stresses and strains expected in this zone.

As far as the material modelling is concerned, the mechanical features have been imposed in terms of true stress-true strain values.

In order to simulate the same boundary conditions of the experimental tests, the nodes belonging to the free edges of one of the two external plates have been rigidly constrained to a reference point with all the degrees of freedom restrained. Nodes belonging to the free edges of the other plate have been tied to a reference point where all the displacements have been restrained except the one perpendicular to the column flange plane. Along this direction, the reference point has been subjected to a controlled displacement linearly increasing up until the attainment of the ultimate strain of the specimen. A standard analysis procedure has been implemented considering both the mechanical and the geometrical non-linearity.

As described in [1], such a model is able to reproduce exactly the experimental evidences and the corresponding force-displacement curves.

## 4. Investigation on the Axial Load Effect

**Figure 4.**
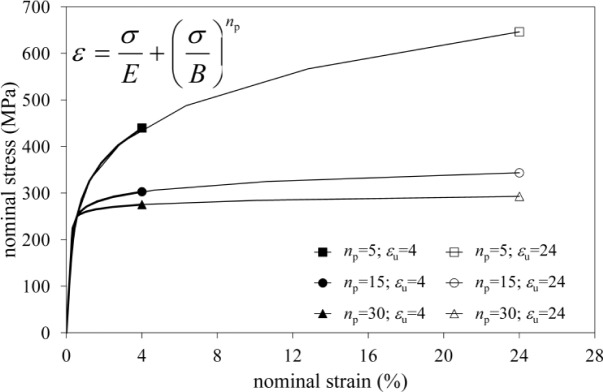
Nominal stress-strain curves for the six constitutive laws assumed in the parametric analysis.

The part of the panel zone under horizontal tensile stresses transmitted by the beam flange could be also subjected to vertical stresses due to the presence of column axial loads. The interaction between these stresses could produce a reduction in tension strength of the column web. In order to investigate this aspect, a parametric study has been carried out. The analysis described in the previous section has been repeated considering different values of axial load on the column. In addition, several materials have been taken into account by imposing Ramberg-Osgood relationships characterized by a unique conventional yielding strength *f*_0_ of 250 MPa, two different ultimate strains (ε_u_ = 4, 24) and three strain hardening parameters (*n*_p_ = 5, 15, 30). In this way, six different material stress-strain relationships, which are depicted in Figure 4, have been examined.

As far as the geometry is concerned, a standard HEM450 column section has been considered. This section, for compression forces, can be classified as Class 1, according to EC9. Thus, it should allow avoiding effects related to local buckling.

The column axial load, both in compression (when negative) and tension, ranges from 0% to 100% of the cross section yielding strength *N*_Rd_, with intermediate values selected every 0.25·*N*_Rd_.

**Figure 5.**
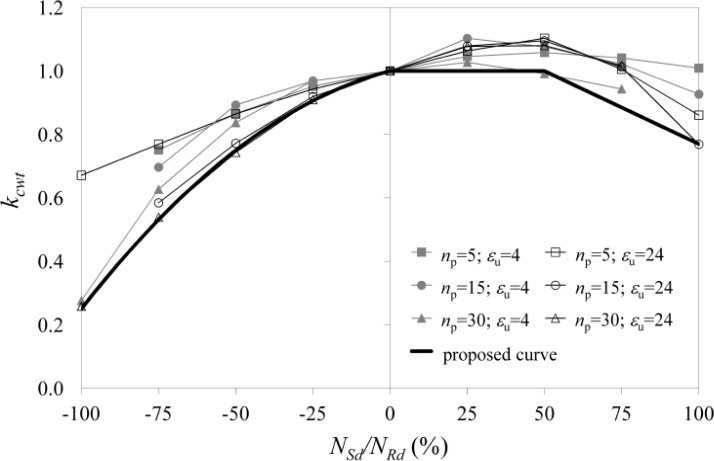
Column web in tension strength–axial load interaction.

The obtained numerical results are reported in Figure 5, where the *k*_cwt_ factor given in Equation (8), namely the ratio between the numerical measure of the component strength *F*_Rd,N_ and the strength given in Equation (4), is expressed as a function of the ratio between the column axial load *N*_sd_ and the corresponding axial strength *N*_Rd_:

$$k_{\text{cwt}}=\frac{F_{\text{Rd,N}}}{F_{\text{Rd},0}}$$ (8)

As expected, when the compressive axial load increases, the column web in tension strength reduces progressively, until the lower bound is reached when *N*_c_ = *N*_Rd_, where 20% of the strength corresponding to *N*_c_ = 0 is still available due to the inelastic resources. On the other hand, the increase of the tensile axial load produces an increase of the component strength due to the Poisson effect, up to axial load values ranging between 0.25·*N*_Rd_ and *N*_Rd_. After such a value, the strength decreases, with minimum values of almost 70% of the strength computed without column axial load.

The lower bound of the factor *k*_cwt_ is enveloped by the bolted curve given in Figure 5, which has been plotted according to Equation (9):

$$\begin{array}{c} k_{\text{cwt}}=-0.5{\left(\frac{N_{\text{Sd}}}{N_{\text{Rd}}}\right)}^{2}+0.25\left(\frac{N_{\text{Sd}}}{N_{\text{Rd}}}\right)+1\text{for }N_{\text{Sd}}\text{/}N_{\text{Rd}}\;\leq 0\%\\ k_{\text{cwt}}=\min\left[1;1.23-0.46\frac{N_{\text{Sd}}}{N_{\text{Rd}}}\right]\text{for }N_{\text{Sd}}\text{/}N_{\text{Rd}}\;>0\% \end{array}$$ (9)

Therefore, the above formulation is conservative and can be considered for implementation in codes and recommendations dealing with aluminium joints.

## 5. Influence of Heat Affected Zones

The presence of heat affected zones (HAZ) is due to aluminium welding procedures that commonly are applied when diverse industrial production technologies, such as die casting, are not carried out. For this reason, a predictive method, able to account for the strength reduction due to HAZ, should be developed. To this purpose, a parametric analysis has been carried out.

**Figure 6.**
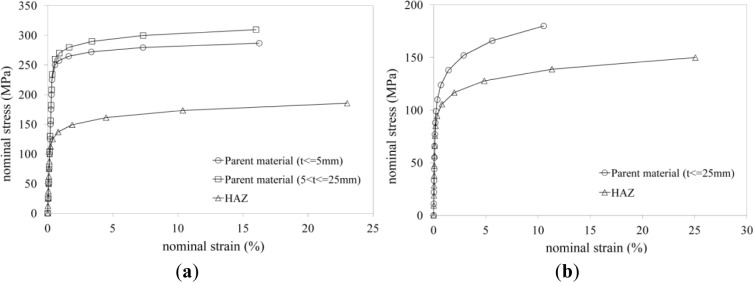
The (**a**) EN-AW 6082-T6; and (**b**) EN-AW 6061-T4 stress-strain constitutive relationship curves.

Considering that the extension of the HAZ depends on the geometry of the welded elements, five different column section profiles have been considered, namely the standard European section I-100, HEA 100, HEB 400, HEM 450 and IPE 300. In addition, a MIG (Metal Inert Gas) welding procedure has been hypothesized. Two commercial alloys have been considered as not affected parent material, namely the EN AW 6082-T6 and the EN AW 6061-T4, whose stress strain relationships have been modelled according to a Ramberg-Osgood law [12], as shown in Figure 6.

The former is characterized by a lower stain hardening and a larger ultimate strain. In the same figure, the mechanical responses of the corresponding heat affected materials are also shown. These have been selected according to EC9 and CNR-DT 208. For the EN AW 6082-T6 heat affected material, it is possible to note a reduction, with respect to the parent material, of 50% and 40%, for the yield and ultimate strength, respectively, an increment of 40% of the ultimate strain and a decrease of the strain hardening parameter *n*_p_ of about 60%.

Also, for the EN-AW 6061-T4 heat affected alloy, it is possible to observe a decrease of both the yielding and ultimate strength with respect to the parent material (14% and 17%, respectively), an increase of the ultimate strain (150%) and a lowering of the strain hardening parameter (20%).

**Figure 7.**
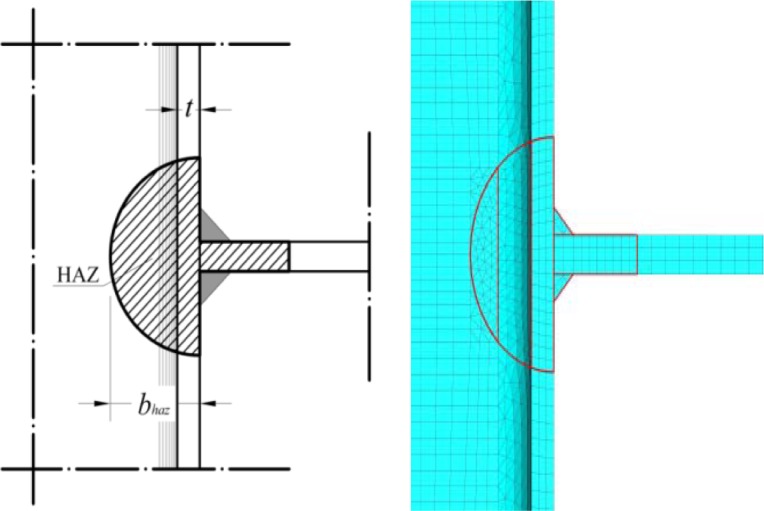
Heat affected zone extent according to Eurocode 9.

The extension of the heat affected zone (*b*_HAZ_) for the two materials has been fixed according to a circular shape detected as a function of the section profile geometry, as prescribed by Eurocode 9 (see Figure 7).

For each section geometry and parent material, three cases of analysis have been run with the same numerical procedure previously presented. For the first case (Case 1), it has been assumed that the whole specimen is made of the parent material only. For the second case (Case 2), parent and heat affected materials have been selected according to the correct stress-strain relationship shown in Figure 6. For the third case (Case 3), it has been considered that the whole specimen is made of the heat affected material only. Therefore, the analysis related to “Case 2” resembles the actual behaviour of the specimen under investigation, while, “Case 1” and “Case 3” are considered as reference limit cases.

**Figure 8.**
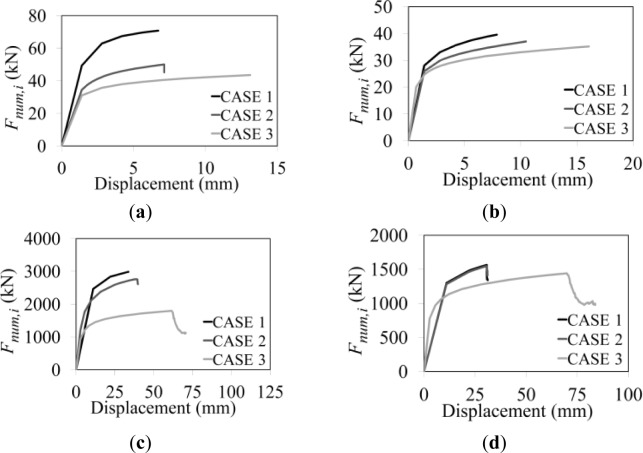

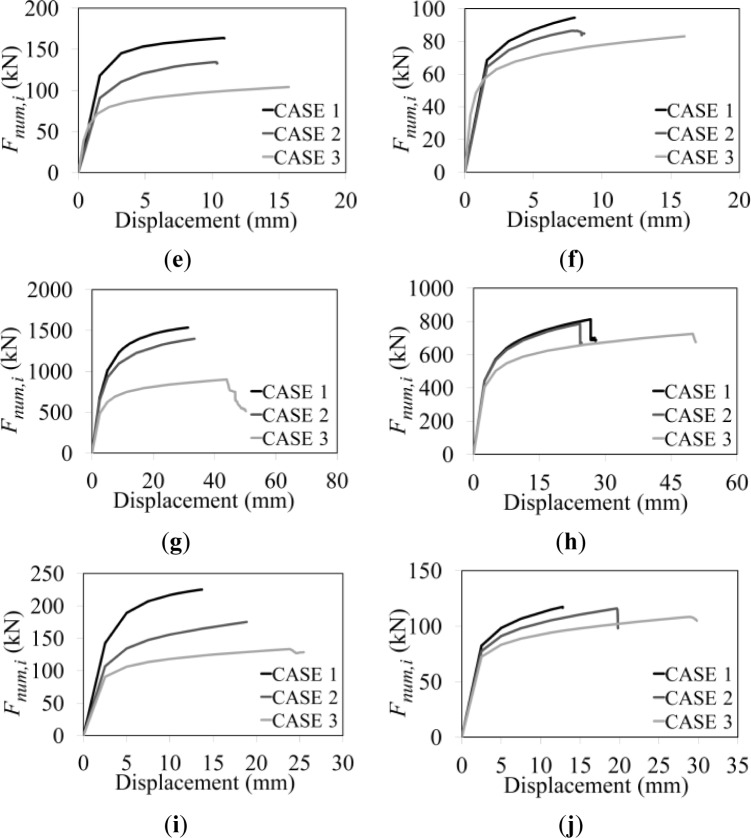
Numerical force-displacement curves: I-100 column section made of (a) 6082-T6 and (**b**) 6061-T4; HEM450 column section made of (**c**) 6082-T6 and (**d**) 6061-T4; HEA100 column section made of (**e**) 6082-T6 and (**f**) 6061-T4; HEB400 column section made of (**g**) 6082-T6 and (h) 6061-T4; IPE200 column section made of (**i**) 6082-T6 and (**j**) 6061-T4.

In Figure 8, the obtained results for the above three cases are provided in terms of Force-Displacement curves.

The obtained outcomes led to finding, for each section and material selected, the relationship given in Equation (10) among the three maximum measured strengths *F*_cwt,Rd,*i*_, namely the value of the forces *F*_num,i_ read for each case, when the ultimate strain is attained.

$$F_{\text{cwt},\text{Rd},2}=F_{\text{cwt},\text{Rd},1}\frac{b_{\text{eff},1}-b_{\text{haz}}}{b_{\text{eff},1}}+F_{\text{cwt},\text{Rd},3}\frac{b_{\text{haz}}}{b_{\text{eff},1}}$$ (10)

In the above equation, *b*_eff,1_ is the effective width for a not-welded specimen (Case 1) computed consistently to Equation (4), whereas *b*_haz_, related to “Case 3” is obtained in the same manner, but accounting for the ductility and the strain hardening characterizing the heat affected material. *F*_cwt,Rd,1_ and *F*_cwt,Rd,3_ represent the strength obtained by Equation (4) assuming the β factor corresponding to the mechanical features of the parent and the heat affected material, respectively.

The reliability of the proposed formulation is shown in Table 1, where the strength obtained by the numerical model *F*_cwt,Rd,2,num_ is normalized to the one given by Equation (9). In the same table, the numerical strength used for Case 1 (*F*_cwt,Rd,1,num_) and Case 3 (*F*_cwt,Rd,3,num_) are normalized to the ones obtained by Equation (4) by using relevant values of β. It is possible to observe that for all three cases almost the same scatter is provided, this meaning that the strength proposed in Equation (10) for welded specimens can be adopted with the same safety level of not-welded specimens (*i.e*., “Case 1”, parent material only).

It has also to be observed that for some profiles the above ratios exceed 1, this meaning that both Equations (4) and (10) could be unconservative. This is due to the fact that Equation (4) has been calibrated on the basis of one cross-section only (I-100), whereas, for different geometry, an additional corrective factor should be adopted in order to account for the effect due to the bending of the column flange, which will be also investigated by the authors. Anyway, considering the wide population of cross-sections considered in the present study, a strength reduction of almost 20% seems to be enough to provide conservative results according to both Equations (4) and (10).

## 6. Conclusions

In this paper, a parametric numerical investigation carried out by means of FE numerical models, reproducing the column web in tension component of aluminium beam-to-column joints, has been presented. It has been evidenced that the presence of a column axial load can reduce significantly the component strength (up to 80% for column in compression and 20% for column in tension).

In order to account for these detrimental effects, a corrective factor *k*_cwt_ to be applied to the strength of the component, in absence of column axial loads, has been presented.

In addition, the strength reduction effect provided the presence of the heat affected zone, in case of welded details, has been investigated. It has been proven that, in the presence of a heat affected zone, the component strength can be expressed as a function of the strengths computed in the case of a component completely made of parent material and a component made of heat affected material.

The obtained outcomes and the related proposed analytical procedure, together with the results shown in previous papers, provide a simple but robust formulation, which could be implemented in codes and guidelines dealing with aluminium joints.

## Figures and Tables

**Table 1.** Numerical *vs.* Analytical (Equation (9)) strength of heat affected aluminium specimens.

Profile	Alloy	*F*_cwt,Rd,2,num_	*F*_cwt,Rd,2_	*F*_cwt,Rd,1_/*F*_cwt,Rd,num,1_	*F*_cwt,Rd,1_/*F*_cwt,Rd,num,1_	*F*_cwt,Rd,2_/*F*_cwt,Rd,2,num_
I-100	EN AW 6082-T6	58	55	1.00	0.99	0.95
HEM 450		2763	2715	0.98	0.94	0.98
HEB 400		1399	1319	0.95	0.93	0.94
HEA 100		134	161	1.19	1.13	1.19
IPE 200		175	191	1.04	1.01	1.09

I-100	EN AW 6061-T4	37	30	0.83	0.85	0.82
HEM 450		1545	1201	0.78	0.78	0.78
HEB 400		786	598	0.75	0.78	0.76
HEA 100		87	81	0.90	0.95	0.94
IPE 200		116	93	0.83	0.83	0.8

## References

[1] (2007). Eurocode 9: Design of Aluminium Structures—Part 1–1: General Structural Rules.

[2] (2011). Istruzioni per la Progettazione, l’Esecuzione ed il Controllo di Strutture di Alluminio.

[3] (2003). Eurocode 3: Design of Steel Structures—Part 1–8: Design of Joints.

[4] De Matteis G., Mandara A., Mazzolani F.M. (2000). T-stub aluminum joints: Influence of behavioural parameters. Comput. Struct.

[5] De Matteis G., Brescia M., Formisano A., Mazzolani F.M. (2009). Behaviour of welded aluminium T-stub joints under monotonic loading. Comput. Struct.

[6] De Matteis G., Naqash M.T., Brando G. (2012). Effective length of aluminium T-stub connections by parametric analysis. Eng. Struct.

[7] Graham J.D., Sherbourne A.N., Khabbaz R.N. (1959). Welded Interior Beam to Column Connections.

[8] Aribert J.M., Lauchal A., Nawawy O.I. (1981). Elastic-Plastic Modelization of the Resistance of a Column in the Compression Region. Constr. Met.

[9] Witteveen J., Stark J.W.B., Bijlaard F.S.K., Zoetemeijer P. (1982). Welded and bolted beam-to-column connections. ASCE J. Struct. Div.

[10] Matusiak M. (1999). Strength and Ductility of Welded Structures in Aluminium Alloys. Ph.D. Thesis.

[11] Sarracco G., Brando G., De Matteis G. Material influence on the strength of aluminium column web in tension.

[12] Ramberg W., Osgood W.R. (1943). Description of Strzess-Strain Curves by Three Parameters.

[13] (1997). ABAQUS, Theory Manual; version 5.7.

